# Knowledge, Attitudes, and Practices of Healthcare Workers on Cervical Cancer Screening in Rural Healthcare Facilities of the Eastern Cape

**DOI:** 10.3390/healthcare13182316

**Published:** 2025-09-16

**Authors:** Ziphelele Ncane, Laston Gonah, Guillermo Alfredo Pulido Estrada, Monwabisi Faleni, Sibusiso Cyprian Nomatshila

**Affiliations:** 1 Department of Public Health, Walter Sisulu University, Mthatha 5117, South Africa; lgonah@wsu.ac.za (L.G.); gpulidoestrada@wsu.ac.za (G.A.P.E.); mfaleni@wsu.ac.za (M.F.); 2 WSU Society and Health Research Institute, Walter Sisulu University, Mthatha 5117, South Africa

**Keywords:** cervical cancer, Papanicolaou test, attitudes, practices, barriers

## Abstract

**Introduction/Objectives:** Cervical cancer screening is a vital preventive strategy, yet the extent of healthcare workers’ knowledge, attitudes, and practices (KAP) can significantly influence its uptake, especially in rural settings. This study aimed to explore the knowledge, attitudes, and practices regarding cervical cancer screening among nurses in selected rural health facilities of the Eastern Cape province, South Africa. **Methods:** A cross-sectional, quantitative study was conducted among 108 nurses selected from 12 health facilities across two districts in the Eastern Cape. All participants had received some training on cervical cancer screening. Structured questionnaires were used to assess knowledge, attitudes, and practices. Comparisons between professional nurses (higher academic qualification) and enrolled nurses (lower academic qualification) were made using appropriate statistical tests. **Results:** Findings revealed significant disparities in knowledge and attitudes between professional and enrolled nurses. Professional nurses demonstrated significantly better knowledge and more positive attitudes towards cervical cancer screening than enrolled nurses, who showed inadequate knowledge (*p* = 0.021) and negative attitudes (*p* = 0.023). Despite universal training, the level of academic qualification remained a key factor influencing KAP. **Conclusions:** Academic qualification is closely linked to knowledge and attitudes regarding cervical cancer screening among nurses. Health workforce policies and programmatic initiatives should prioritize targeted training for enrolled nurses, focusing on addressing specific knowledge and skill gaps. Tailored interventions are recommended to enhance competencies and improve cervical cancer screening practices among all nursing cadres.

## 1. Introduction

Cervical cancer (CaCx) is a significant global health challenge, particularly in low- and middle-income countries (LMICs), where it is the second leading cause of cancer mortality among women [1,2,3]. An estimated 444,500 new cases and 230,200 deaths occur annually in LMICs [4,5]. The global cancer burden is projected to increase, with 22.2 million new cases and 12.7 million cancer-related deaths expected by 2030 [2]. LMICs will bear a disproportionate burden, accounting for over half of new cases and two-thirds of cancer-related deaths, due to limited access to early diagnosis and quality care [3,4,5].

The World Health Organization (WHO) has called for action to eliminate cervical cancer as a public health issue by achieving an incidence rate of less than 4 per 100,000 women-years [1,6]. WHO recommends that countries implement basic cancer control aspects through strategies such as HPV DNA self-sampling to improve cervical cancer screening in rural areas [7]. Expanding prevention, diagnosis, and treatment services and integrating cancer care into primary healthcare can help address existing gaps and optimize resource use. However, access to basic screening, such as the Papanicolaou test (Pap smears), is limited, particularly in LMICs [3].

In South Africa, cervical cancer remains a major public health concern, with the Eastern Cape Province experiencing higher incidence and mortality rates [4,7]. Roughly 74% of people acknowledge cervical cancer as a serious public health concern, but routine screening procedures are not prompted by this awareness [4,8]. Merely 27.7% of healthcare professionals say they have screened for cervical cancer. Less than half of these employees have received training in screening techniques or interpreting Pap smear results, which is the reason for this disparity [5]. Effective screening programs in rural regions with low resources are hampered by this lack of skills. In addition, current programs usually lack quality assurance systems, and many nurses perform screens infrequently, despite their crucial role in healthcare delivery [9]. High rates of cervical cancer morbidity and death in rural areas are a result of this disconnect, which jeopardizes the early detection and treatment of cervical precancerous lesions [1]. Despite government efforts to provide cervical cancer screening through provincial and district hospitals, access to screening services in rural areas is severely limited by geographic and resource constraints [1]. The province’s limited resources further exacerbate the cervical cancer burden, impacting healthcare infrastructure, staffing, and service availability [10].

In rural communities, primary healthcare clinics staffed by nurses serve as the main point of care, providing early cancer diagnosis and improving survival outcomes [5]. These nurses typically fall into two main categories, namely, enrolled nurses (nursing diploma holders) and registered nurses (nursing degree holders). However, existing research suggests that primary healthcare professionals in South Africa may lack sufficient knowledge and skills regarding cervical cancer prevention and treatment, potentially compromising their quality of care [5,6]. This study sought to examine the knowledge, attitudes, and practices of nurses regarding cervical cancer screening in rural healthcare facilities of the Eastern Cape province in South Africa. Identifying gaps in knowledge and practices can inform strategies to improve cervical cancer screening and care in resource-constrained settings.

## 2. Materials and Methods

### 2.1. Study Design

This was a cross-sectional study.

### 2.2. Setting

The study was conducted at 12 selected facilities in OR Tambo and Alfred Nzo districts in the Eastern Cape Province, which are Mntwana Clinic, Mount Frere Gateway Clinic, Dr Malizimpehle Gateway Clinic, Mthatha Gateway Clinic, St Barnabas Gateway Clinic, Mhlakulo CHC, Qumbu CHC, Mqanduli CHC, Mbekweni CHC, Baziya CHC and Isilimela Hospital.

### 2.3. Population

The study population comprised professional nurses and enrolled nurses working at specified health institutions in the OR Tambo and Alfred Nzo districts of the Eastern Cape province. These were selected due to their typical direct involvement in cervical cancer screening and management in the province.

### 2.4. Sampling Procedure and Sample Size Calculation

Purposive sampling was used to select the study participants among consenting health professionals responsible for cancer screening in 12 health facilities in the OR Tambo and Alfred Nzo districts. The sample size was calculated using the following formula:

$$n=\frac{Z_{\propto }^{2}\times P(100-P)}{e^{2}}$$

$$n=\frac{{\left(1.96\right)}^{2}\times 50(100-50)}{10^{2}}=\frac{3.84\times 2500}{100}=96$$

where expected proportion: P = 50%

95% confidence level: (Z_α_ = 1.96)

Margin of error (e) = 10%

A sample size of 96 healthcare workers directly involved in cervical cancer screening and management was required for the study, where 20% was added to account for non-response and confounding bias, giving a total sample size of 108 participants.

### 2.5. Data Collection

A validated structured self-administered questionnaire [11] was used to collect data. The questions were adopted from a standardized and validated tool for use in the community cancer screening outreach (BMSF Cancer Symptom Screening Tool, version 2.1; 27 August 2020) (see Table S1). Key variables assessed for knowledge included awareness of screening guidelines; knowledge of screening methods; understanding of risk factors, signs and symptoms; and interpretation of screening results. Assessment of attitudes included questions on perceived importance and effectiveness of cervical cancer screening, perceived confidence in screening procedures, perceived barriers, and attitudes towards patient education. Regarding practices, the study assessed whether the participants discussed screening procedures with patients, referral and follow-up practices, screening procedures, and practices related to patient education in screening.

Data was collected by the researcher alone to ensure consistency and was validated using structured and interviewer-administered questionnaires. The questionnaire collected information on respondents’ demographic characteristics, knowledge, attitudes, and practices related to cervical cancer and cervical cancer screening. The questionnaire consisted of 30 items, distributed evenly across three domains: knowledge, attitudes, and practices. Knowledge questions covered risk factors, symptoms, and screening guidelines. Attitude items explored beliefs and perceptions regarding cervical cancer and screening. Practice questions assessed self-reported screening-related behaviors.

Eligible participants were approached during staff meetings and provided with detailed information about the study’s purpose, procedures, and confidentiality measures. Written informed consent was obtained prior to questionnaire administration. Participation was voluntary, with no financial compensation provided.

### 2.6. Data Analysis

Data entry and management were conducted using Microsoft Excel, while data analysis was performed using the Statistical Package for the Social Sciences (SPSS) software, version 29 (IBM SPSS Statistics). Results were presented according to demographics, knowledge, attitudes, and practices, and were displayed in tables as frequencies (n) and proportions (%). Logistic regression was used to examine the relationship between selected predictor variables and the level of knowledge (inadequate vs. adequate). Associations were tested using a 95% confidence interval, and a *p*-value of less than 0.05 was considered statistically significant.

### 2.7. Validity and Reliability

The study used validated questions from other studies to ensure accuracy and consistency. To ensure the reliability and relevance of the research instrument, a pilot study was conducted among ten participants who were subsequently excluded from the study. The data collection tool was revised according to pre-test findings prior to its implementation.

### 2.8. Variable Measurement

Knowledge, attitudes, and practices were assessed using 30 questions: that is, 10 questions for each, corresponding to a maximum of 10 points (100%). Knowledge was rated as adequate or inadequate, attitudes as positive or negative, and practices as either good or bad, based on participant responses. The total score for each category of knowledge, attitudes, and practices was dichotomised using percentiles, where the 70th percentile was the cut-off value. Participants who scored above the 70th percentile (7 out of 10 or 70%) were coded “1”, representing adequate knowledge, positive attitudes, or good practices. Those participants scoring below the 70th percentile were coded “0”, representing inadequate knowledge, negative attitudes, or bad practices.

## 3. Results

### 3.1. Demographic Information

A total of 108 nurses responsible for providing cervical cancer services participated in the study (Table 1). Most (91.7%) of the participants were females, and the mean participant age was 41.7 years (SD ± 10.78 years). Most of the participants (89.8%) were registered nurses, while the remaining 10.2% were enrolled nurses.

Knowledge with regard to cervical cancer screening was assessed against nurses’ demographic characteristics, including their knowledge regarding cervical cancer screening (Table 2). A total of 51 (47.2%) demonstrated inadequate knowledge and 57 (52.8%) demonstrated adequate knowledge of cervical cancer screening. Being a registered nurse (a higher academic qualification) was associated with adequate knowledge, compared to enrolled nurses (a lower academic qualification) who demonstrated inadequate knowledge (*p* = 0.015) of cervical cancer screening. Nurses’ age group, occupation, years of experience, and prior exposure to specialized training on cervical cancer screening and management were not associated with their knowledge.

Logistic regression on characteristics of nurses influencing their knowledge of cervical cancer screening and management consistently found that a higher academic qualification is significantly associated with adequate knowledge (*p* = 0.021; OR = 7.04; 95%CI: 1.34–36.91) of cervical cancer screening among nurses (Table 3). Age group, years of practice, and prior exposure to specialized training on cervical cancer screening were not statistically significant predictors of knowledge in this sample (*p* > 0.05).

### 3.2. Participants’ Attitudes Toward Cervical Cancer Screening

In logistic regression, professional nurses were more likely to have positive attitudes compared to enrolled nurses (Table 4). Age group, years of practice, and prior exposure to specialized training on cervical cancer screening were not statistically significant predictors of attitudes in this sample (*p* > 0.05).

Regarding practices, none of the assessed sociodemographic and personal characteristic predictors were significantly associated with cervical cancer screening practices (*p* > 0.05). This was mainly due to a small proportion of participants who scored poorly on practice (5.6%), which was inadequate to determine any association. Special training refers to targeted education and capacity-building programs that equip nurses with the knowledge, skills, and competencies needed to prevent, detect, and manage cervical cancer. These programs may include workshops, short courses, or in-service training [12].

## 4. Discussion

Findings from this study indicate that adequate knowledge of and positive attitudes toward cervical cancer screening among the participating nurses were both independently influenced by higher academic qualifications, despite the common specialized training background or prior universal exposure to specialized training on cervical cancer screening. Related practices were not influenced by any of the studied predictor variables, such as participant demographics, age group, or years of professional experience, as most participants reported good screening practices.

In concurrence with findings from this study, existing evidence shows that advanced education can enhance positive understanding and foster positive attitudes toward cervical cancer screening [4,11,13]. Higher education is known to improve critical thinking skills and understanding of screening-related concepts [14]. It also increases one’s confidence in making informed decisions when following screening protocols, compared to those with lower academic qualifications [14,15].

The inconsistency in study findings—where practices were not significantly influenced by any of the studied factors, yet concomitant differences in knowledge and attitudes were based on academic qualifications—may have been influenced by several factors. In light of the findings, it could have been scientifically plausible if inadequate knowledge and negative attitudes were associated with bad practices, and good practices were associated with adequate knowledge and positive attitudes [4,11,13,16]. The actual screening practices may have been influenced by factors beyond those studied, such as health facility-related factors (e.g., availability of resources) and patient-related factors (e.g., compliance), thereby leading to the observed discrepancy [15]. From another perspective, previous studies found that healthcare workers can still exhibit good practices, despite having inadequate knowledge and negative attitudes due to routine or protocol-driven behavior influenced by guidelines, protocols, and standard operating procedures [15,16]. External motivators or requirements, such as supervision, audits, performance evaluations, and workplace culture or norms, can also enforce strict adherence to screening practices [16]. Again, the measurement of practices was based on self-reported responses, rendering the collected data prone to recall and social desirability biases. The reported practices may not have been the actual practices undertaken by the participants.

Future studies must consider more objective ways to determine attitudes and practices beyond self-reported data, such as through observational studies, chart reviews or audits, performance metrics or indicators, behavioral assessments or simulations, and peer review or supervisor evaluations, triangulating the approaches where possible [17,18].

## 5. Conclusions

In conclusion, the findings highlight the need for targeted training interventions to enhance cervical cancer screening competencies among nurses, particularly those with lower academic training, such as enrolled nurses. To inform effective training programs, policies, and strategies in rural settings, it is essential to tailor training content to address specific knowledge and skill gaps, incorporate felt needs and context-specific challenges, and prioritize capacity-building initiatives that support nurses in delivering high-quality cervical care, ultimately contributing to the reduction in disparities in rural areas.

## Figures and Tables

**Table 1:** Socio-demographic distribution.

Demographic Variables		No.	%
Age group	20–29	17	15.7
	30–39	32	29.6
	40–49	32	29.6
	50–59	21	19.4
	60 and above	6	5.6
Total		108	100.0
Gender	Male	9	8.3
	Female	99	91.7
Total		108	100.0
Location	Baziya CHC	17	15.7
	Isilimela hospital	16	14.8
	Qumbu CHC	15	13.9
	Mthatha Gateway Clinic	13	12
	Mbekweni CHC	13	12
	Mount Frere Gateway Clinic	11	10.2
	Dr Malizompehle Gateway Clinic	9	8.3
	Mhlakulo CHC	6	5.6
	Mntwana Clinic	6	5.6
	Mqanduli CHC	1	0.9
	St Barnabas Gateway Clinic	1	0.9
Total		108	100.0
Marital status	Never married	52	48.1
	Married	39	36.1
	Cohabiting	2	1.9
	Divorced	9	8.3
	Widowed	6	5.6
Total		108	100.0

**Table 2:** Association between nurses’ demographic characteristics and knowledge of cervical cancer screening.

Variables		Knowledge				Total		*p* Value
		Inadequate		Adequate n (%)				
		n	%	n	%	n	%	
Age group	20–29	7	13.7	10	17.5	17	15.7	0.840
	30–39	17	33.3	15	26.3	32	29.6	
	40–49	13	25.5	19	33.3	32	29.6	
	50–59	11	21.6	10	17.5	21	19.4	
	60 and above	3	5.9	3	5.3	6	5.6	
Total		51	100.0	57	100.0	108	100.0	
Occupation	Registered nurse	42	82.4	55	96.5	97	89.8	0.015
	Enrolled nurse	9	17.6	2	3.5	11	10.2	
Total		51	100	57	100	108	100	
Years of experience	1–5	13	25.5	20	35.1	33	30.6	0.521
	6–10	21	41.2	22	38.6	43	39.8	
	More than 10	17	33.3	15	26.3	32	29.6	
Total		51	100.0	57	100.0	108	100.0	
Special training	Yes	17	33.3	27	47.4	44	40.7	0.138
	No	34	66.7	30	52.6	64	59.3	
Total		51	100.0	57	100.0	108	100.0	

**Table 3:** Logistic regression on the association between personal characteristics and knowledge of cervical cancer and screening.

Predictor	B	SE	OR (95% CI)	*p*-Value
Age Group (Ref: 20–29)				
30–39	−0.157	0.688	0.85 (0.22–3.29)	0.819
40–49	0.583	0.754	1.79 (0.41–7.85)	0.44
50–59	0.176	0.843	1.19 (0.23–6.22)	0.835
60 and above	−0.204	1.16	0.82 (0.08–7.92)	0.861
Occupation (Ref: Professional Nurse)				
Enrolled Nurse	1.952	0.845	7.04 (1.34–36.91)	0.021
Years of Practice (Ref: 1–5 years)				
6–10 years	−0.482	0.575	0.62 (0.20–1.91)	0.402
>10 years	−1.071	0.692	0.34 (0.09–1.33)	0.122
Special Training (Ref: Yes)				
No	−0.757	0.45	0.47 (0.19–1.13)	0.092

**Table 4:** Association between sociodemographic characteristics and attitudes toward cervical cancer screening among nurses (n = 108).

Variable	Positive Attitudes		Negative Attitudes		Total		*p*-Value
	n	%	n	%	n	%	
**Age group**							0.137
20–29	17	19.1	0	0.0	17	15.7	
30–39	25	28.1	7	36.8	32	29.6	
40–49	24	27.0	8	42.1	32	29.6	
50–59	17	19.1	4	21.1	21	19.4	
≥60	6	6.7	0	0.0	6	5.6	
**Occupation**							0.023 *
Professional Nurse	83	93.3	14	73.7	97	89.8	
Enrolled Nurse	6	6.7	5	26.3	11	10.2	
**Years of Practice**							0.424
1–5 years	29	32.6	4	21.1	33	30.6	
6–10 years	33	37.1	10	52.6	43	39.8	
More than 10 years	27	30.3	5	26.3	32	29.6	
**Special Training**							0.371
Yes	38	42.7	6	31.6	44	40.7	
No	51	57.3	13	68.4	64	59.3	

* Fischer’s exact test.

## Data Availability

The datasets generated and/or analyzed during the study are not publicly available due to privacy and ethical restrictions protecting the confidentiality of study participants. However, anonymized data may be made available from the corresponding author upon reasonable request and subject to approval by the Walter Sisulu University ethics committee. The data used in this study are available upon request from Z. Ncane, Email: zncane@wsu.ac.za.

## Supplementary Materials

The following supporting information can be downloaded at: https://www.mdpi.com/article/10.3390/healthcare13182316/s1.
