# Supplementary material for: Knowledge, Attitudes, and Practices of Healthcare Workers on Cervical Cancer Screening in Rural Healthcare Facilities of the Eastern Cape
Source: Healthcare (Basel). 2025 Sep 16;13(18):2316. doi: 10.3390/healthcare13182316 (PMC12469757; doi:10.3390/healthcare13182316)
Supplement: Supplementary file 1 [file healthcare-13-02316-s001.zip › healthcare-3794451-supplementary.pdf]

Table S1

Knowledge, Attitude, Practices and Barriers on Cervical Cancer Among Health Workers Responsible for Cancer Services in the Eastern Cape Province

SECTION 1: Interview Information		
1.1	Name of Health Facility	
1.2	Date of interview (dd/Month/ YYYY)	
1.3	Full name of interviewer	

SECTION 2: Demographic characteristics of the respondent			
2.1	Biological sex	*1. Male*	◻
		2. Female	◻
2.2	Date of birth (dd/month/yyyy)		
2.3	Nursing Education	________________________________	
2.5	Marital status	*1. Never Married*	◻
		2. Married (including lobola)	◻
		3. Cohabiting	◻
		4. Divorced	◻
		5. Widowed	◻
		6. Separated	◻
2.6	Occupation	*1. Registered (Professional) Nurse*	◻
		2. Enrolled Nurse	◻
2.7	Length of practice (years)	____________________	
2.8	Department	____________________	

SECTION 3: Knowledge assessment of Cervical Cancer (Tick the correct answer)			
3.1	What is the primary cause of cervical cancer?<br>*[key: Human papillomavirus (HPV)]*		
3.2	Which of the following is a risk factor for cervical cancer (tick all appropriate) –<br>*[Key: All of the above]*	a. Early age of sexual intercourse<br>b. Multiple sexual partners<br>c. Smoking<br>*d.* All of the above	☐<br>☐<br>☐<br>☐
3.3	Which screening method is most used for cervical cancer?<br>*[Key: Pap smear]*	a. Pap smear<br>b. HPV DNA testing<br>c. Visual inspection with acetic acid (VIA)<br>d. Colposcopy	☐<br>☐<br>☐<br>☐
3.4	What is the recommended age range for cervical cancer screening?<br>*[Key: 21-65 years]*	a. 21-65 years<br>b. 18-50 years<br>c. 30-60 years<br>d. 40-70 years	

3.5	What is the purpose of HPV vaccination in relation to cervical cancer?<br>*[Key: b]*	a. To treat cervical cancer<br>b. To prevent HPV infection and reduce cervical cancer risk<br>c. To diagnose cervical cancer<br>d. To screen for cervical cancer
3.6	According to South Africa's cervical cancer screening policy, how often should women with normal Pap smear result undergo screening?<br>*[Key: d]*	a. Once every year<br>b. Every 2-3 years<br>c. Every 5 years<br>d. Every 10 years starting at age 30
3.7	Which of the following is a symptom of advanced cervical cancer<br>*[key: d]*	a. Abnormal vaginal bleeding<br>b. Pelvic pain<br>c. Back pain<br>d. All of the above
3.8	What is the role of colposcopy in cervical cancer screening?<br>*[Key: c]*	a. To confirm diagnosis of cervical cancer<br>b. To screen for cervical cancer<br>c. To evaluate abnormal Pap smear results<br>d. To treat cervical cancer
3.9	How does HIV Infection affect cervical cancer risk?<br>*[Key: a]*	a. Increases risk<br>b. Decreases risk<br>c. No effect<br>d. unknown
3.10	What is the goal of cervical cancer screening programs?<br>*[Key: b]*	a. To diagnose cervical cancer at an early stage<br>b. To prevent cervical cancer through HPV vaccination<br>c. To treat cervical cancer<br>To reduce morbidity and mortality from cervical cancer

**SECTION 4: Attitude assessment of Cervical Cancer** *[Key: Yes]*			
4.1	I believe cervical cancer screening is important for women's health.	a. Yes<br>b. No	☐<br>☐
4.2	I feel confident in my ability to counsel patients about cervical cancer screening	a. Yes<br>b. No	☐<br>☐
4.3	Cervical cancer screening is a priority in my clinical practice	a. Yes<br>b. No	☐<br>☐
4.4	I am comfortable discussing cervical cancer screening with patients.	*a. Yes*<br>*b. No*	☐<br>☐
4.5	Cervical cancer screening guidelines are clear and easy to follow.	*1. Yes*	☐
		2. No	☐
4.6	I believe cervical cancer screening saves lives	*1. Yes*	☐
		2. No	☐
4.7	I feel that cervical cancer screening is a worthwhile investment in healthcare resources.	*1.Yes*	☐
		2. No	☐
4.8	I am confident in my ability to perform cervical cancer screening.	*1. Yes*	☐
		2. No	☐
4.9	Have you ever been screened for cervical cancer?	*1. Yes*	☐
		2. No	☐
4.10 I believe patient education is key to increasing cervical cancer screening uptake			1. Yes<br>2. No

**SECTION 5: Practices and Barriers of cervical cancer care**			
5.1	Do you counsel patients about cervical cancer screening?<br>*[Key: a]*	a. Yes<br>b. No	
5.2	How often do you perform cervical cancer screening in your clinical practice?<br>*[Key: a,b]*	a. Always<br>b. Most of the time<br>c. Sometimes<br>d. Rarely<br>e. Never	☐<br>☐<br>☐<br>☐<br>☐
5.3	Do you follow established guidelines (e.g. national, ACS, USPSTF, ASCCP for cervical cancer screening?<br>*[Key: a]*	a. Yes<br>b. No	
5.4	What screening method do you most commonly use for cervical cancer?<br>[Key: a]	a. Pap smear<br>b. HPV DNA testing<br>c. VIA<br>d. Colposcopy<br>e. Others, specify	☐<br>☐<br>☐<br>☐
5.5	How do you stay up to date with cervical cancer screening information and practices?<br>*[Key: a-c]*	a. Regularly read medical literature<br>b. Attended conferences/works hops<br>c. Online/onsite training/guidline<br>d. None	☐<br>☐<br>☐<br>☐
5.6	How do you determine the frequency of cervical cancer screening for your patients?<br>*[Key: b]*	a. Based on patient risk factors<br>b. According to established guidelines<br>c. Based on patient preference<br>d. Other (please specify)	☐<br>☐<br>☐<br>☐
5.7	DO you provide HPV vaccination recommendations to your patients?<br>*[Key: a]*	a. Yes<br>b. No	☐<br>☐
5.8	How do you document Cervical cancer screening results in patients' records?<br>*[Key: a-c]*	a. Electronically<br>b. Papper-based<br>c. Both<br>d. Other (specify)	☐<br>☐<br>☐<br>☐
5.9	Do you participate in quality control measures for cervical cancer screening (e.g., proficiency testing, peer review)?<br>[Key: a]	a. Yes<br>b. No<br>c. Sometimes	☐<br>☐<br>☐
5.10	How confident are you in your ability to perform cervical cancer screening?<br>[Key: a]	a. Confident<br>b. Not confident	☐<br>☐
